# Deciphering the genomic and lncRNA landscapes of aerobic glycolysis identifies potential therapeutic targets in pancreatic cancer

**DOI:** 10.7150/ijbs.49243

**Published:** 2021-01-01

**Authors:** Li-Li Zhu, Zheng Wu, Rong-Kun Li, Xin Xing, Yong-Sheng Jiang, Jun Li, Ya-Hui Wang, Li-Peng Hu, Xu Wang, Wei-Ting Qin, Yong-Wei Sun, Zhi-Gang Zhang, Qin Yang, Shu-Heng Jiang

**Affiliations:** 1 State Key Laboratory of Oncogenes and Related Genes, Ren Ji Hospital, School of Biomedical Engineering, Shanghai Jiao Tong University, Shanghai 200240, P.R. China.; 2 State Key Laboratory of Oncogenes and Related Genes, Shanghai Cancer Institute, Ren Ji Hospital, School of Medicine, Shanghai Jiao Tong University, Shanghai 200240, P.R. China.; 3 Department of Radiation Oncology, Ren Ji Hospital, School of Medicine, Shanghai Jiao Tong University, Shanghai 200127, PR China.; 4 Institute of Oncology, Affiliated Hospital of Jiangsu University, Zhenjiang 212001, P.R. China.; 5 The Fengxian Hospital, Southern Medical University, Shanghai 201499, PR China.; 6 Department of Biliary-Pancreatic Surgery, Ren Ji Hospital, School of Medicine, Shanghai Jiao Tong University, Shanghai 200217, P.R. China.

**Keywords:** Tumor metabolism, Energy metabolism, LncRNA, CNVs, FEZF1-AS1

## Abstract

Aerobic glycolysis, also known as the Warburg effect, is emerged as a hallmark of most cancer cells. Increased aerobic glycolysis is closely associated with tumor aggressiveness and predicts a poor prognosis. Pancreatic ductal adenocarcinoma (PDAC) is characterized by prominent genomic aberrations and increased glycolytic phenotype. However, the detailed molecular events implicated in aerobic glycolysis of PDAC are not well understood. In this study, we performed a comprehensive molecular characterization using multidimensional ''omic'' data from The Cancer Genome Atlas (TCGA). Detailed analysis of 89 informative PDAC tumors identified substantial copy number variations (*MYC*, *GATA6*, *FGFR1*, *IDO1,* and *SMAD4*) and mutations (*KRAS*, *SMAD4*, and *RNF43*) related to aerobic glycolysis. Moreover, integrated analysis of transcriptional profiles revealed many differentially expressed long non-coding RNAs involved in PDAC aerobic glycolysis. Loss-of-function studies showed that LINC01559 and UNC5B-AS1 knockdown significantly inhibited the glycolytic capacity of PDAC cells as revealed by reduced glucose uptake, lactate production, and extracellular acidification rate. Moreover, genetic silencing of LINC01559 and UNC5B-AS1 suppressed tumor growth and resulted in alterations in several signaling pathways, such as TNF signaling pathway, IL-17 signaling pathway, and transcriptional misregulation in cancer. Notably, high expression of LINC01559 and UNC5B-AS1 predicted poor patient prognosis and correlated with the maximum standard uptakevalue (SUVmax) in PDAC patients who received preoperative^ 18^F-FDG PET/CT. Taken together, our results decipher the glycolysis-associated copy number variations, mutations, and lncRNA landscapes in PDAC. These findings improve our knowledge of the molecular mechanism of PDAC aerobic glycolysis and may have practical implications for precision cancer therapy.

## Introduction

Pancreatic ductal adenocarcinoma (PDAC) is a highly lethal malignancy with an overall 5-year survival rate of < 8%. PDAC is predicted to become the second leading cause of cancer-related deaths by the year 2030 and is refractory to most therapeutic strategies 1. The deep whole exome sequencing study of PDAC have identified key mutations and somatic copy number alterations (SCNAs) in many key oncogenes and tumor suppressor genes, including *KRAS*, *TP53*, *CDKN2A*, and *SMAD4* 2, 3. Unfortunately, none of these genetic drivers are currently targetable, thus making it difficult to develop effective treatment modality for PDAC.

PDAC is characterized by prominent desmoplastic reaction and poor vascularity, which led to a nutrient-deficiency and hypoxic tumor microenvironment 4. Energy metabolism is extensively reprogrammed in PDAC to enable cell survival and proliferation under this hostile condition. One of the most common metabolic alterations of cancer cells is aerobic glycolysis, also known as Warburg effect, which provides cancer cells with sufficient intermediary metabolites for generation of reducing equivalents and macromolecules (nucleotides) required for rapid proliferation and to avoid apoptosis 5, 6. Aerobic glycolysis can be regulated by many oncogenic signals, such as MYC, HIF-1α, and PI3K/AKT pathway 7-9. Recently, emerging evidence suggests that long non-coding RNA (lncRNA) plays crucial roles in a variety of cellular processes, such as chromatin remodeling, embryonic development, cell differentiation, energy metabolism, and tumorigenesis by regulating gene expression through multiple mechanisms 10, 11. Several dysregulated lncRNAs with oncogenic activities have been identified in PDAC, such as LINC00673, FAM83H-AS1, and GLS-AS 12, 13. However, the lncRNAs that responsible for PDAC aerobic glycolysis remain largely unknown.

In this study, by leveraging large-scale PDAC genomic data and molecular profiles from The Cancer Genome Atlas (TCGA) cohort, we revealed many copy number variations, mutations, and lncRNAs related to aerobic glycolysis in PDAC. Two aberrantly expressed lncRNAs, LINC01559 and UNC5B-AS1, were demonstrated to regulate PDAC aerobic glycolysis and tumor growth. Thus, this study, 1) reveals a molecular link between genomic alteration and cancer metabolism, 2) broadens understanding of lncRNA-mediated regulatory roles of aerobic glycolysis in PDAC, and 3) provides potential therapeutic targets for PDAC treatment.

## Materials and Methods

### Bioinformatic analysis

The genomic and level 3 molecular profiling data of the PDAC patients were downloaded from The Cancer Genome Atlas (TCGA) database (http://cancergenome.nih.gov). Copy number variation was assessed from the Affymetrix genome-wide human SNP array 6.0 platform using GISTIC2.0 (Version 2.0.22). Somatic mutations, single-nucleotide variants (SNVs), small insertion, and deletion were determined by Mutect. Fractions of single nucleotide substitutions in the six possible mutation classes (ie, C>T, C>A, C>G, A>G, A>C, and A>T) were calculated for each sample. Tumor mutational burden (TMB) was defined as the number of somatic, coding, base substitution, and indel mutations per mega base of genome examined. The nonparametric Mann-Whitney U-test was used to determine the significance in TMB difference between two populations. The R software package Limma was used to identify differentially expressed genes. Gene set enrichment analysis was performed using the GSEA software. Gene ontology and pathway analyses were performed with DAVID (https://david.ncifcrf.gov/).

### Cell lines, culture conditions, and transfection

Pancreatic cancer cell lines (AsPC-1, BxPC-3, Capan-1, PANC-1, and SW1990) were all preserved in Shanghai Cancer Institute. Mycoplasma contamination was tested and cell characterization was performed using polymorphic short tandem repeat (STR) profiling. Cells were cultured in RPIM-1640 or DMEM (Life Technologies, USA) supplemented with 10% fetal bovine serum (Gibco, Grand Island, NY, USA). All cells were cultured at 37°C in a saturated humidity atmosphere containing 5% CO_2_. For cell transfections, specific siRNA targeting human LINC01559 and UNC5B-AS1 along with control-siRNA targeting no known gene sequence were synthesized from GenePharma (Shanghai, China). All transfections were conducted using Lipofectamine® RNAiMAX reagent (ThermoFisher Scientific, #13778030) following the manufacturer's instructions. The sequence information for siRNAs are as follow: si-LINC01559-#1, GCACCCAACAUGUUGGAUAdTdT; si-LINC01559-#2, GCCCUAAAUGUGGUUGGAUdTdT; si-UNC5B-AS1-#1, GAUCCUGCCUCAGGGAAAUdTdT; si-UNC5B-AS1-#2, GCCUUCCGCAAAGUGUUCUdTdT. Moreover, the same targeting sequences of si-LINC01559-#1 and si-UNC5B-AS1-#1 were used for generation of stable knockdown cell lines. In brief, BxPC-3 were transfected with recombinant lentivirus-transducing units in the presence of polybrene (Sigma, 5 μg/ml). One week later, the stable sh-LINC01559 or sh-UNC5B-AS1 cells were selected by 2 μg/ml puromycin (Sangon, Shanghai, China).

### Real-time quantitative PCR

Total RNA from PDAC cells was extracted using the Trizol reagent (Invitrogen, USA) and reverse-transcribed to cDNA using PrimeScript RT-PCR kit (Takara, Japan) according to the manufacturer's instructions. Quantitative real-time PCR was performed with SYBR *Premix Ex Taq* (Takara, Japan) using the Applied Biosystems ViiA7 machine. The primers sequences are as follow: LINC01559 forward, 5'-TCTGAAACGAAGGGCTGACC-3'; LINC01559 reverse, 5'-TCTACGAGCGCTCTGACTCT-3'; UNC5B-AS1 forward, 5'-GATCCTGCCTCAGGGAAA-3'; UNC5B-AS1 reverse, 5'-GCTCAAGAGGTTGGGACT-3'; β-actin forward, 5'-CATGTACGTTGCTATCCAGGC-3'; β-actin reverse, 5'-CTCCTTAATGTCACGCACGAT-3'. Relative expression level of each gene was calculated using the 2^(-ΔΔCt)^ method and normalized to β-actin gene. Experiments were repeated at least three times.

### Immunohistochemical (IHC) analysis

IHC staining was performed as reported previously described 14. In brief, paraffin-embedded tumor tissue sections were deparaffinized and rehydrated with graded ethanol. Endogenous peroxidase was blocked by 0.3% hydrogen peroxide in methanol. Antigen retrieval was done in 10 mM citrate buffer (pH 6.0) at 100°C for 15 minutes, followed by incubation with 10% BSA (Sangon, Shanghai, China) for 1 h at room temperature. After washing with phosphate-buffered saline (PBS) for three times, the slides were incubated with primary antibody against Ki67 (Cell Signaling Technology, #9449, USA) at 4°C overnight. The next day, slides were incubated with HRP (rabbit) second antibody and the immunoreactivity was generated by DAB substrate liquid (GeneTech, Shanghai, China). Finally, sections were counterstained by hematoxylin.

### Measurement of glucose and lactate

Glucose consumption and lactate production were tested using a Glucose Assay kit (Sigma, MAK181) and a Lactate Assay kit (BioVision, K607-100) as described previously 15. The values were normalized to total protein concentration of each sample. The experiment was performed in triplicate and repeated twice.

### Measurement of extracellular acidification rate

Extracellular acidification rate (ECAR) was monitored with XF96 Extracellular Flux Analyzer (Seahorse Bioscience) according to the manufacturer's instructions. BxPC-3 cells were seeded in a XF96-well plate at a density of 1 × 10^4^ per well the day before determination. Cells were plated in XF96 Cell Culture Microplates (Seahorse Bioscience) at an initial cellular density of 1 × 10^4^ cells/well. One hour before, the culture medium was replaced by seahorse buffer, which is consists of DMEM, phenol red, 25 mM glucose, 2 mM sodium pyruvate, and 2 mM glutamine. Then, ECAR was determined by a sequential injection of 10 mM glucose, 1 μM oligomycin, and 50 mM 2-deoxyglucose (Agilent Technologies, #103017). ECAR in each well was normalized to total protein content. Each assay was run in triplicate.

### Fluorescence *in situ* hybridization (FISH) experiment

RNA-FISH was performed using Fluorescent *in situ* Hybridization Kit (Servicebio Company, technology CO., LTD, Wuhan, China). LINC01559 and UNC5B-AS1 probes were designed and synthesized by Servicebio Company and labeled with Digoxin (DIG). Paraffin sections (5 μm) of human PDAC tumor tissues were deparaffinized, rehydrated with graded ethanol, and subjected to digestion with proteinase K (20 μg/ml), followed by incubation with hybridization buffer supplemented with FISH probe and washed with PBS for three times. Anti-DIG secondary antibodies were used to detect the signals, and DAPI was applied to stain the nuclei. Fluorescence signal detection was performed using aconfocal laser scanning microscope (Leica, Germany). All patients included in this study signed informed consent and this study was approved by the Institutional Review Board of Ren Ji Hospital, School of Medicine, Shanghai Jiao Tong University.

### Colony formation assay

Single-cell suspension was plated at a density of 1,000 cells per plate in 6-well plates. The culture medium was changed every 3 days. After 10-14 days, the colonies were washed twice with PBS, fixed with 10% methanol, and stained with 0.25% crystal violet (Beyotime, C0121). Colonies with more than fifty cells were counted under a microscope.

### RNA sequencing analysis

RNA-sequencing experiment was performed to identify the potential molecular mechanism. In brief, total RNA from sh-Ctrl, sh-LINC01559 or sh-UNC5B-AS1 BxPC-3 cells was extracted by Trizol. RNAseq was performed by Sinotech Genomics (Shenzhen, China). Gene expression was calculated using FPKM method. The edgeR software package was used to analyze the difference in gene expression between groups. Multiple hypothesis test corrections were performed after calculating the p-value. The threshold of p-value is determined by controlling false discovery rate (FDR). Kyoto Encyclopedia of Genes and Genomes (KEGG) was used for enrichment of differentially expressed genes.

### Animal experiment

Nude mice (male, 6-week old) were used for subcutaneous xenograft experiment. Mice were maintained under a specific pathogen-free condition with free access to food and water. All experimental procedures were approved by the Research Ethics Committee of East China Normal University. Mice received subcutaneous injections of 1 × 10^6^ sh-Ctrl, sh-LINC01559 or sh-UNC5B-AS1 BxPC-3 cells. Four weeks later, all mice were sacrificed, and tumor tissues were isolated and tumor weight was determined.

### Statistical analysis

Statistical analyses were conducted using R/Bioconductor packages, SPSS version 18 (SPSS, Inc., Chicago, IL), and GraphPad Prism 7 (version 5.04, La Jolla, CA). Quantitative data are expressed as means ± SD. Log rank test and Kaplan-Meier curves were used to analyze the survival distributions. Correlation analysis was determined using the Spearman's test. A two tailed *t* test was used to identify significant differences incomparisons unless otherwise stated. Statistical significance was defined as a *P* value less than 0.05.

## Results

### Consensus clustering identifies PDAC glycolysis status

**Figure 1:**
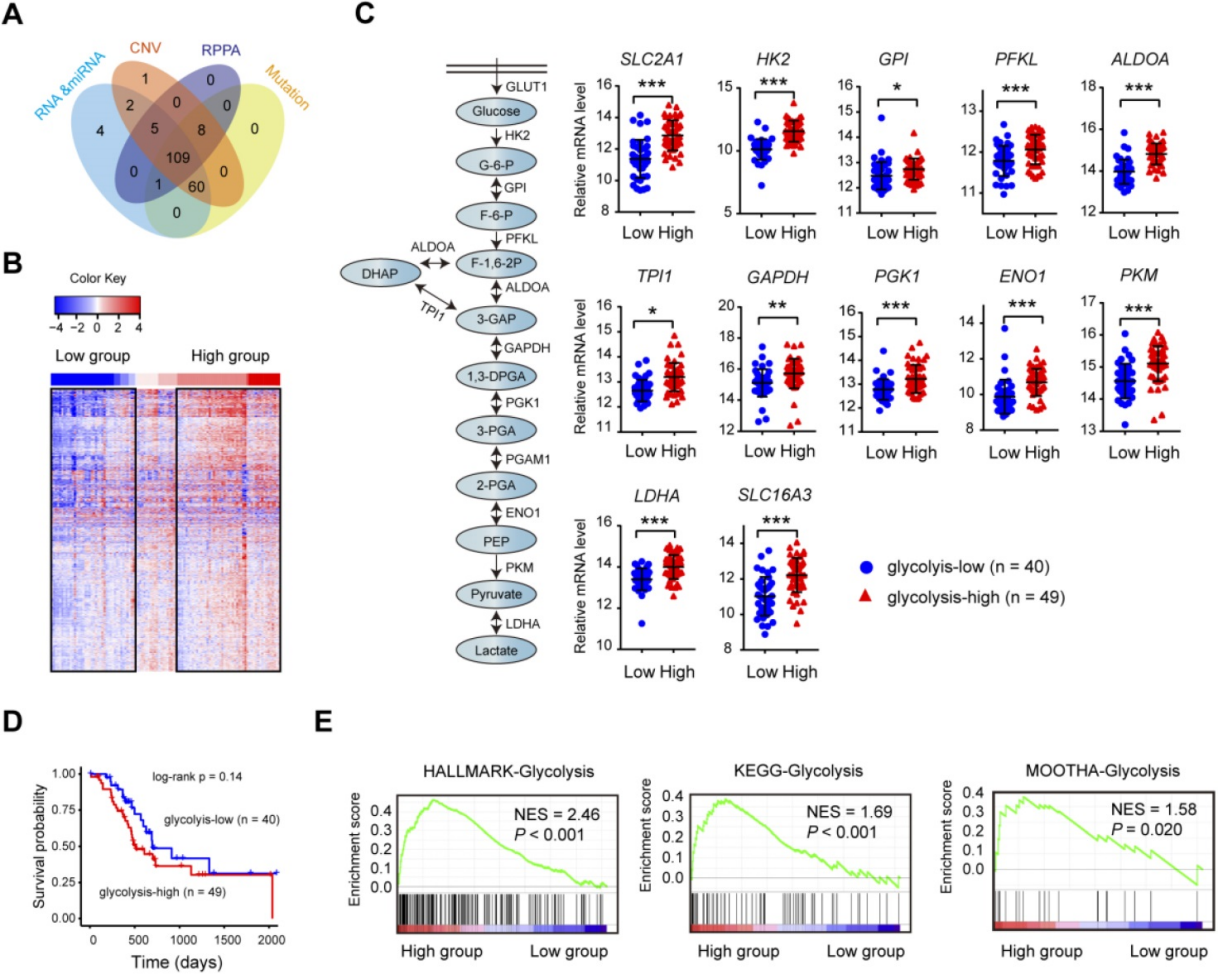
** Consensus clustering identifies PDAC glycolysis status. (A)** PDAC patients (n = 109) with genomic and molecular profiling data were selected for grouping analysis. **(B)** Heat maps of 109 PDAC samples clustered in glycolysis-low and glycolysis-high groups. **(C)** Expression comparison of glycolytic genes withinthe glycolysis-low and glycolysis-high groups. **(D)** Kaplan-Meier curve analysis (log-rank test) of the survival rate between glycolysis-low and glycolysis-high groups. **(E)** Gene set enrichment analysis (GSEA) on three independent glycolysis gene sets across the glycolysis-low and glycolysis-high samples. NES, normalized enrichment score (NES); false discovery rate (FDR) was set at 0.25. *p < 0.05; **p < 0.01; ***p < 0.001.

The matched DNA mutations, copy number alterations, expression profiles of mRNA and lncRNA data on 109 clinically-annotated PDAC were obtained from The Cancer Genome Atlas (TCGA) data portal and subjected for further analysis (**Fig. 1A**). Unsupervised hierarchical cluster was performed in PDAC samples using K-mean equal to 12 and euclidean distance metrics. Clusters containing the 12 glycolysis-signature transcripts (*SLC2A1*, *HK2*, *GPI*, *PFKP*, *ALDOA*, *ALDOC*, *PGK1*, *ENO1*, *ENO2*, *PKM*, *LDHA*, and *SLC16A3*) were used for resampling-based hierarchical clustering of the same samples using ConsensusClusterPlus v.1.16.0 (**Fig. 1B**). The consensus clustering led to the identification of two transcriptional PDAC subtypes: glycolyis-low (n = 40) and glycolysis-high (n = 49) (**Supplementary Table 1**). Expectedly, many key glycolytic components including *SLC2A1*, *HK2*, *PKM*, and *LDHA* had significantly elevated mRNA expression level in the glycolysis-high subtype (**Fig. 1C**). Despite no statistical difference was found, patients in glycolysis-high group showed a poor prognosis compared with the glycolyis-low group (**Fig. 1D**). Gene set enrichment analysis (GSEA) showed that three independent glycolysis gene expression signatures (Hallmark, KEGG and MOOTHA) were consistently enriched in the glycolysis-high groups (**Fig. 1E**). To validate the confidence of this classification approach, we further performed similar analysis using two independent data sets from Gene Expression Omnibus (GEO). As a result, the result obtained from TCGA cohort was also reproducible in GSE15471 and GSE16515 (**Supplementary Fig. 1**). Collectively, the above findings indicate that our classification model was built on meaningful data in the context of aerobic glycolysis.

### Glycolysis-related gene copy number variations in PDAC

**Figure 2:**
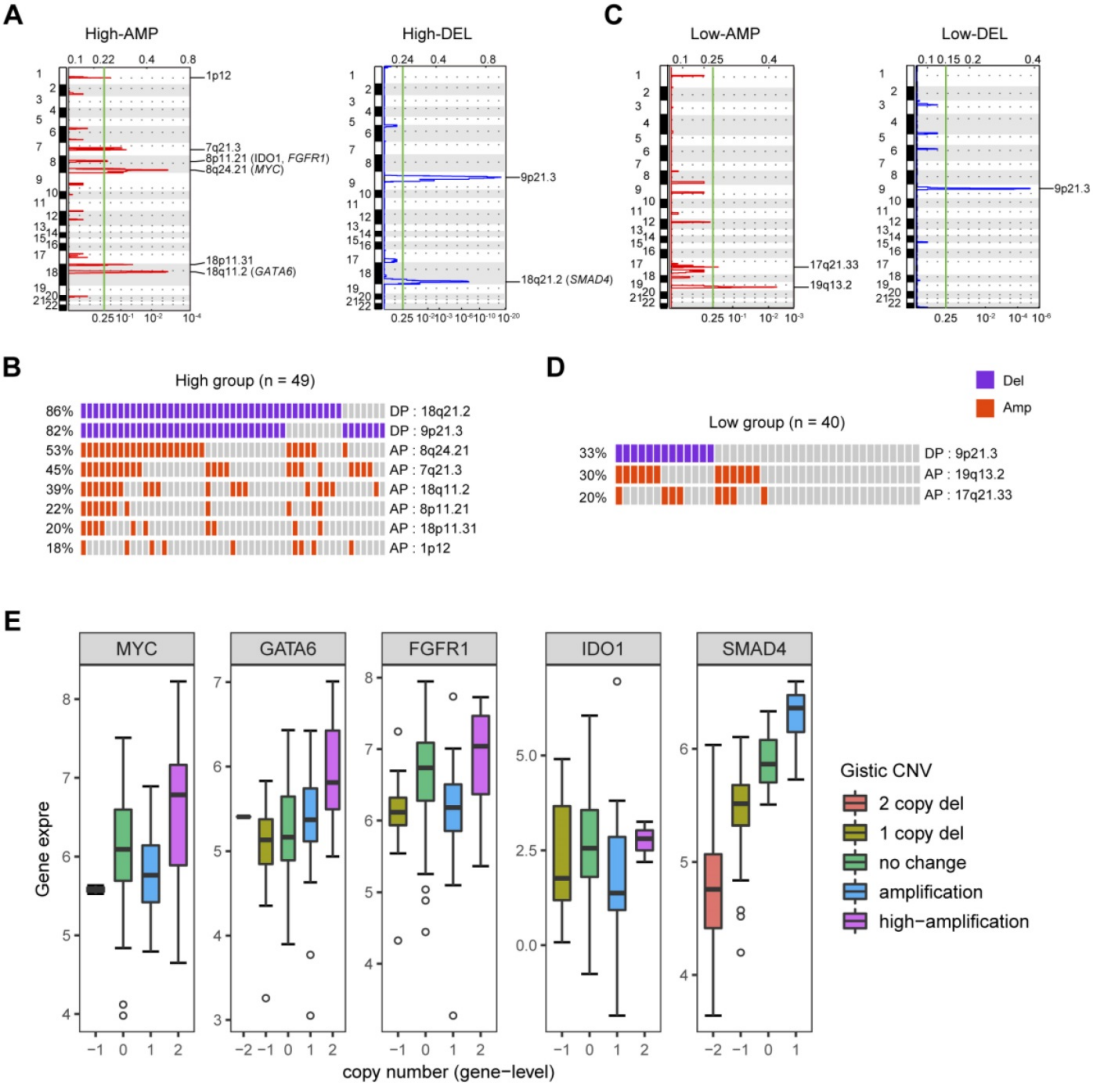
** CNVs related to PDAC glycolysis. (A)** Specific copy number profiles (gains in red and losses in blue) for glycolysis-high PDAC samples. **(B)** Significant CNVs from the glycolysis-high group. Each rectangle represents a PDAC subject. **(C)** Specific copy number profiles (gains in red and losses in blue) for glycolysis-low PDAC samples. **(D)** Significant CNVs from the glycolysis-low group. Each rectangle represents a PDAC subject. **(E)** Association of CNVs and gene expression in *MYC*, *GATA6*, *FGFR1*, *IDO1*, and *SMAD4*.

To identify gene copy number variations (CNVs) related to PDAC glycolysis, we compared the SNP microarray data of glycolysis-high samples to those with lower glycolysis. Significant focal gains and deletions (q < 0.25) were identified in the majority of PDAC samples. In detail, amplifications of 1p12 (18%), 7q21.3 (45%), 8p11.21 (21%), 8q24.21 (20%), 18p11.31 (20%), and 18q11.2 (39%) along with deletions of 9p21.3 (82%) and 18q21.2 (86%) were enriched in the glycolysis-high samples (**Fig. 2A and 2B**). In contrast, amplifications of 19q13.2 (30%) and 17q21.33 (20%) along with deletions of 9p21.3 (33%) were distributed in glycolysis-low samples (**Fig. 2C and 2D**). GISTIC analysis showed a number of recurrent events containing known oncogenic drivers in the glycolysis-high group. These include amplifications of *MYC* (8q24.2), *GATA6* (18q11.2), *FGFR1* (8p11.21), and *IDO1* (8p11.21) as well as deletion of *SMAD4* (18q21.2) (**Fig. 2A; Supplementary Table 2-5**). Integrated analysis showed that copy number variations in *GATA6* and *SDMA4* were closely associated with their gene expression level in PDAC (**Fig. 2E**).

### Glycolysis-related gene mutations in PDAC

**Figure 3:**
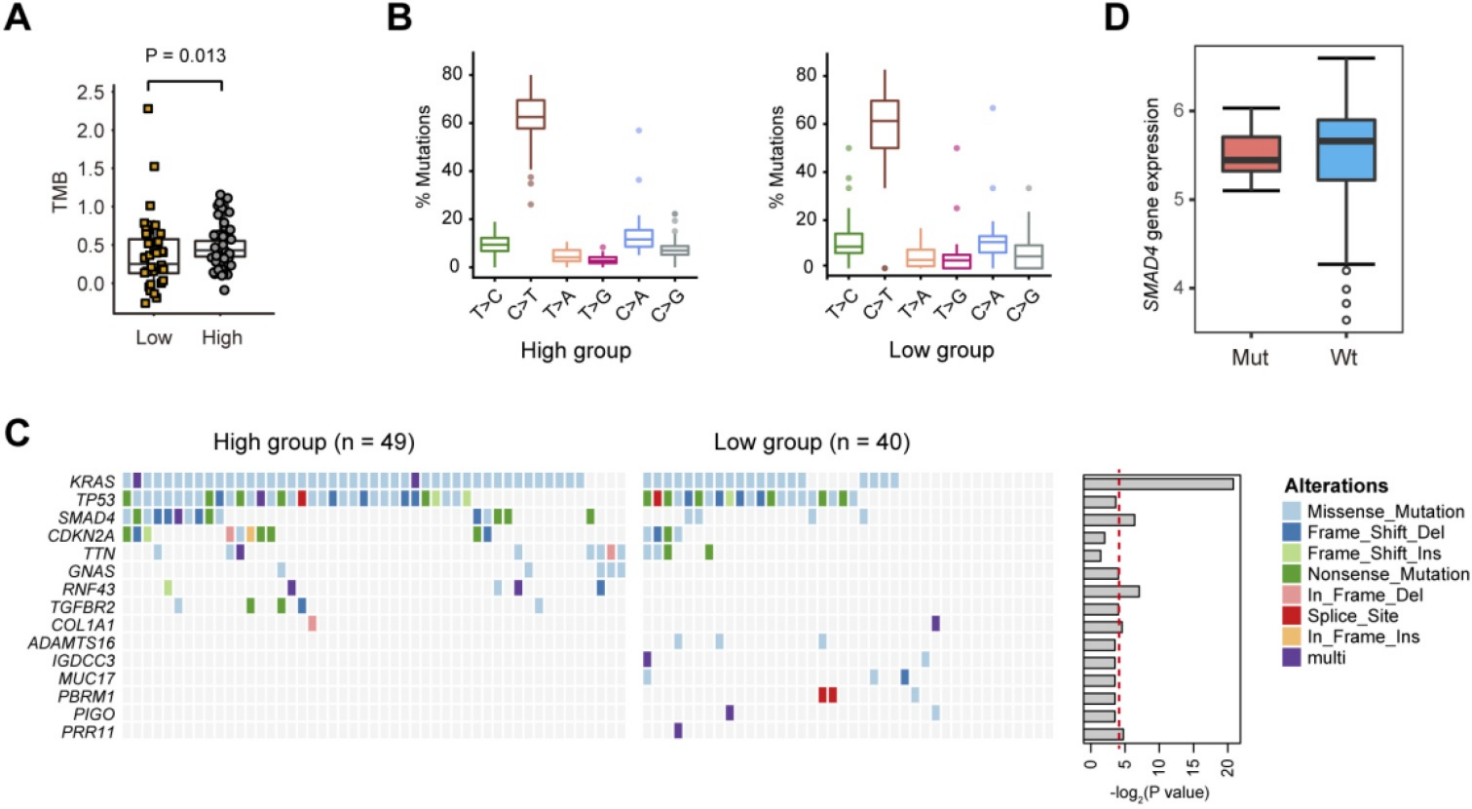
** Gene mutations related to PDAC glycolysis. (A)** The tumor mutation burden (TMB) withinthe glycolysis-low and glycolysis-high groups. **(B)** The percentage of six substitution subtypes (C>A, C>G, C>T, T>A, T>C, and T>G) within the glycolysis-low and glycolysis-high groups. **(C)** Oncoprint of the frequently mutated genes related to PDAC glycolysis.

Next, we evaluated the somatic mutational signatures in the 89 PDAC samples to identify significantly recurring mutations implicated in PDAC glycolysis. As a result, we found a higher total mutation burden (TMB) in glycolysis-high samples compared with that in the glycolysis-low group (**Fig. 3A**; Wilcoxon rank sum test, P = 0.013). No significant difference in the mutation type frequency between the two groups was noticed (**Fig. 3B**). Consistent with previous reports, significant recurrent mutations were identified in *KRAS* (73.0%), *TP53* (61.8%), *SMAD4* (21.3%), and *CDKN2A* (15.7%). In these genes, *KRAS* and *SMAD4* mutations were significantly enriched in glycolysis-high samples (**Fig. 3C**). Notably, mutations in *RNF43* gene (5/49, 10.2%), *GNAS* gene (5/49, 10.2%) and *TGFBR2* gene (5/49, 10.2%) were specifically distributed in the glycolysis-high group. Moreover, mutations in *ADAMTS16* (3/40, 7.5%), *MUC17* (3/40, 7.5%), *IGDCC3* (2/40, 5.0%), *PBRM1* (3/40, 7.5%) and *PIGO* (2/40, 5.0%) genes were exclusively present in the glycolysis-low group (**Fig. 3C**). In PDAC, *KRAS* mutations have been well documented to be essential for anabolic glucose metabolism 16. Loss of SMAD4 enhances PDAC glycolysis by inducing PGK1 upregulation 17. However, mutations in *SMAD4* did not confer a significant effect on the expression level of SMAD4 in PDAC (**Fig. 3D**).

### LncRNAs related to PDAC glycolysis

**Figure 4:**
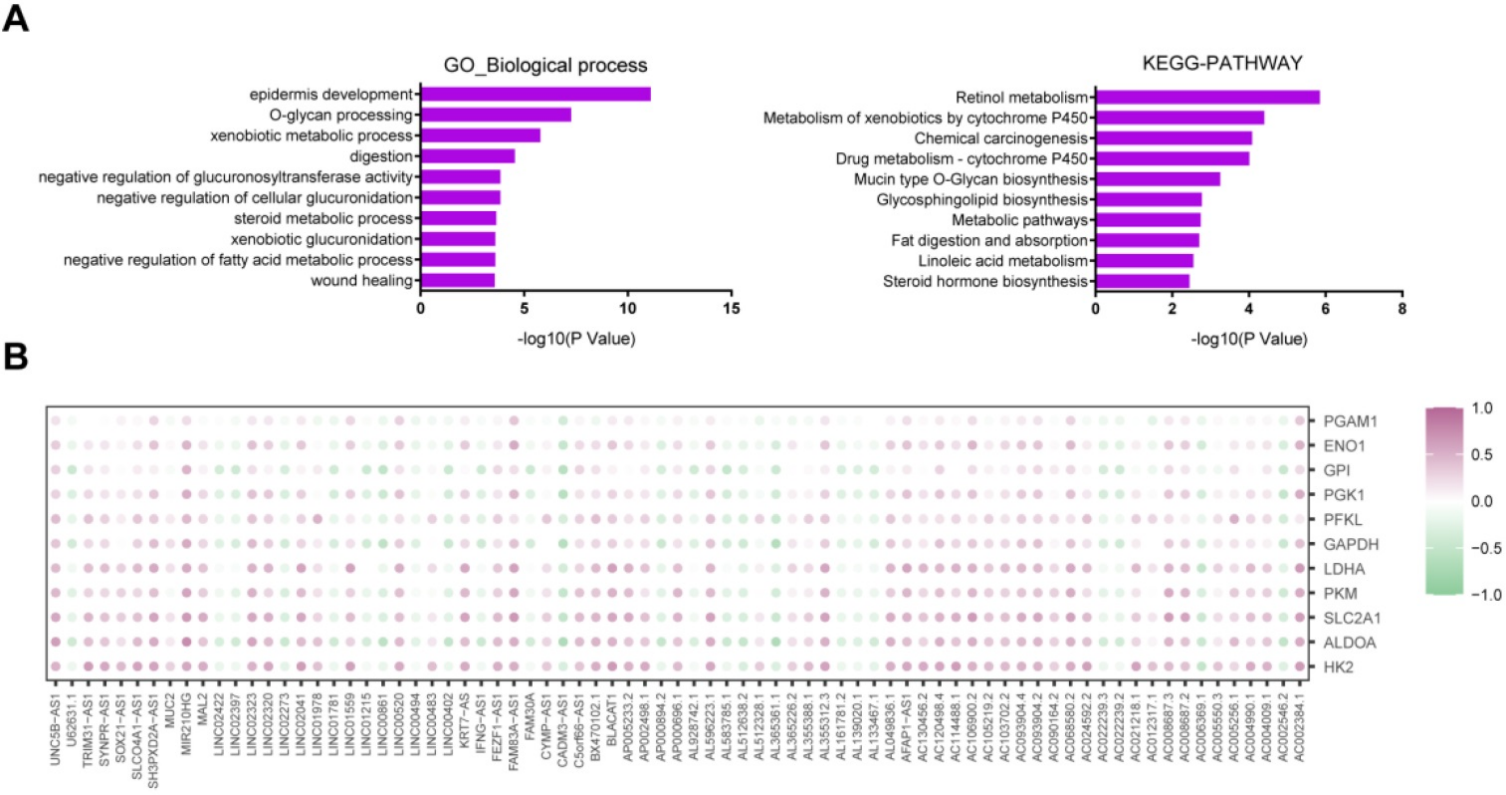
** LncRNAs related to PDAC glycolysis. (A)** GO and KEGG analysis of glycolysis-related differentially expressed genes. **(B)** Expression correction analysis between all of the differentially expressed lncRNAs and glycolytic genes (HK2, ALDOA, SLC2A1, PKM, LDHA, GAPDH, PFKL, PGK1, GPI, ENO1, and PGAM1) in PDAC. Correlation was determined using the Spearman's test.

Significant transcriptional alterations were observed between the glycolysis-high and glycolysis-low groups. As expected, differentially expressed mRNAs were significantly enriched in metabolism-related pathways as revealed by GO and KEGG analysis (**Fig. 4A**). By comparing the RNA sequencing data, we identified 53 significantly up-regulated and 24 down-regulated lncRNAs with a log_2_ (fold change) lager than 2 or less than -2 (**Supplementary Fig. 2 and Table 1**). By correlation analysis, we found that most of these lncRNAs had a close correlation with glucose transporter SLC2A1 and glycolytic enzymes (HK2, ALDOA, PKM, LDHA, GAPDH, PFKL, PGK1, GPI, ENO1, and PGAM1) (**Fig. 4B**).

### Role of LINC01559 and UNC5B-AS1 in PDAC glycolysis

**Figure 5:**
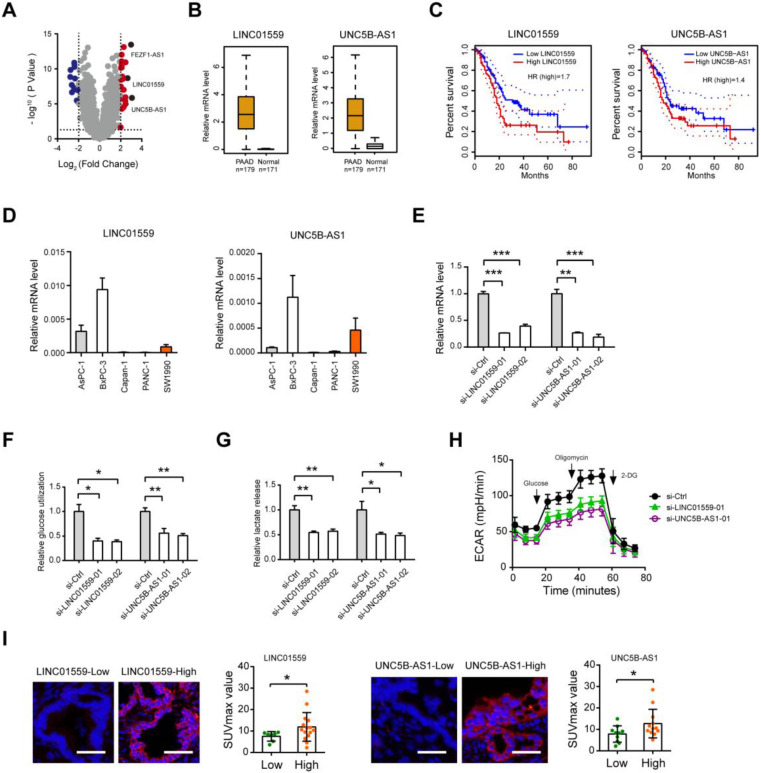
** Regulatory role of LINC01559 and UNC5B-AS1 in PDAC glycolysis. (A)** Volcano plot showed thedifferentially expressed lncRNAs between glycolysis-high and glycolysis-low group. **(B)** TCGA and GTEx database data showed the expression level of LINC01559 and UNC5B-AS1 in PDAC and their normal counterparts. **(C)** Kaplan-Meier analysis showed the overall survival of PDAC patients based on LINC01559 and UNC5B-AS1 expression. **(D)** Real-time qPCR analysis showed LINC01559 and UNC5B-AS1 expression level in PDAC cell lines. **(E)** Real-time qPCR analysis of siRNA-mediated knockdown efficiency of LINC01559 and UNC5B-AS1 in BxPC-3 cells. **(F-H)** Quantification of glucose uptake (**F**), lactate production (**G**), and extracellular acidification rate (**H**) in si-LINC01559, si-UNC5B-AS1, and si-Ctrl BxPC-3 cells. **(I)** Representative photographs FISH analysis in PDAC patients with preoperative^ 18^F-FDG PET/CT scans; scale bar: 50 µm. The correlation between LINC01559 or UNC5B-AS1 expression and the SUVmax was analyzed. *p < 0.05; **p < 0.01; ***p < 0.001.

Among the identified lncRNAs, FEZF1-AS1, LINC01559 and UNC5B-AS1 were the top 3 reported lncRNAs (**Fig. 5A**). Moreover, FEZF1-AS1 has been demonstrated to promote the glycolytic phenotypes of colorectal cancer by regulating PKM2 signaling 18. From the therapeutic point of view, we therefore verified the roles of LINC01559 and UNC5B-AS1, which have been implicated in several oncogenic processes but not involved in glycolysis 19-23. Data from the TCGA + GTEx portal showed that LINC01559 and UNC5B-AS1 were highly expressed in the tumor tissues (n = 179) compared with normal pancreas samples (n = 171) (**Fig. 5B**). Kaplan-Meier curve analysis revealed that elevated expression of LINC01559 and UNC5B-AS1 predicts a poor prognosis in PDAC patients (**Fig. 5C**). To determine whether LINC01559 and UNC5B-AS1 regulate PDAC glycolysis, we performed loss-of-function study in BxPC3 cells, which show higher endogenous level of LINC01559 and UNC5B-AS1 (**Fig. 5D**). Two specific siRNAs against LINC01559 and UNC5B-AS1 efficiently blocked their expression level (**Fig. 5E**). Notably, LINC01559 and UNC5B-AS1 significantly inhibited the glycolytic activity of BxPC-3 cells as demonstrated by reduced glucose utilization (**Fig. 5F**), lactate production (**Fig. 5G**), and extracellular acidification rate (**Fig. 5H**). Moreover, in a cohort of 22 PDAC patients who received preoperative^18^F-FDG PET/CT, we found that the SUVmax was considerably higher in specimens with high LINC01559 or UNC5B-AS1 expression than that in the low expression group (**Fig. 5I**). LINC01559 and UNC5B-AS1 expression were also closely correlated with the mRNA level of many glycolytic components in the glycolysis pathway (**Supplementary Fig. 3**), suggesting a regulatory role of LINC01559 and UNC5B-AS1 in PDAC glycolysis.

### Genetic silencing of LINC01559 and UNC5B-AS1 inhibits tumor growth in PDAC

**Figure 6:**
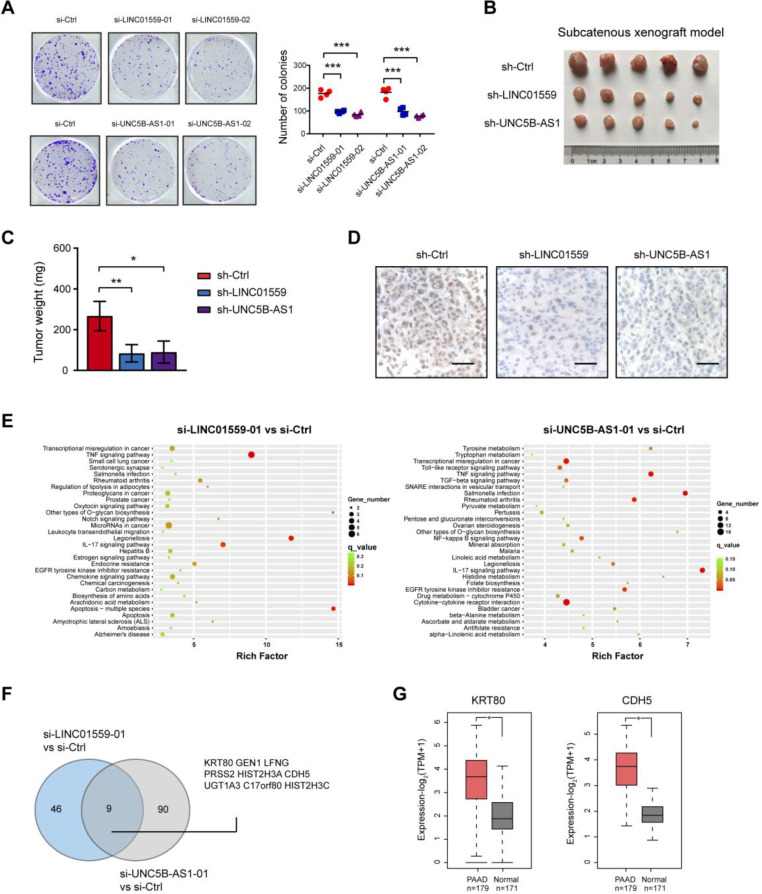
** Genetic silencing of LINC01559 and UNC5B-AS1 inhibits tumor growth in PDAC. (A)** The effect of LINC01559 or UNC5B-AS1 knockdown on BxPC-3 cell proliferation was measured by plate colony formation assay. **(B)** A subcatenous xenograft model showed the effect of LINC01559 or UNC5B-AS1 knockdown on the *in vivo* tumor growth of PDAC (n=5). **(C)** Measurement of tumor weight in sh-Ctrl, sh-LINC01559 andsh-UNC5B-AS1 groups. **(D)** IHC analysis of Ki67 in sh-Ctrl, sh-LINC01559 and sh-UNC5B-AS1 tumor tissues. **(E)** KEGG enrichment of differentially expressed genes upon LINC01559 or UNC5B-AS1 knockdown in BxPC-3 cells. **(F)** Venn diagram showed differentially expressedgenes upon LINC01559 and UNC5B-AS1 knockdown in BxPC-3 cells. **(G)** TCGA and GTEx database data showed the expression level of KRT80 and CDH5 in PDAC and their normal counterparts. *p < 0.05; **p < 0.01; ***p < 0.001.

Increased aerobic glycolysis provides abundant cellular buildings to favor rapid cancer cell proliferation 24. In this study, we revealed that either LINC01559 or UNC5B-AS1 knockdown resulted in significant downregulation in anchorage-dependent growth of PDAC cells (**Fig. 6A**). To test the *in vivo* effect of LINC01559 and UNC5B-AS1 knockdown on tumor growth, a subcatenous xenograft model was generated. As a result, stably knockdown of LINC01559 or UNC5B-AS1 significantly retarded tumor burden as evidenced by tumor weight and the proliferation index Ki67 (**Fig. 6B-D**). Moreover, we performed RNA sequencing to identify the gene expression profiles altered by LINC01559 and UNC5B-AS1. KEGG enrichment analysis of differentially expressed showed that LINC01559 or UNC5B-AS1 knockdown contributed to alterations in several signaling pathways, such as TNF signaling pathway, IL-17 signaling pathway, and transcriptional misregulation in cancer (**Fig. 6E**). Comparative analysis revealed that 9 differentially expressed genes were consistently downregulated by LINC01559 or UNC5B-AS1 knockdown (**Fig. 6F** and **Supplementary Table 6-7**), including genes that have been involved in the diverse oncogenic processes but not previously reported to be related to glycolysis. Notable genes include KRT80 and CDH5 25, 26, which are suspected to function as tumor promoters in human cancers and are highly expressed in PDAC (**Fig. 6G**).

## Discussion

**Figure 7:**
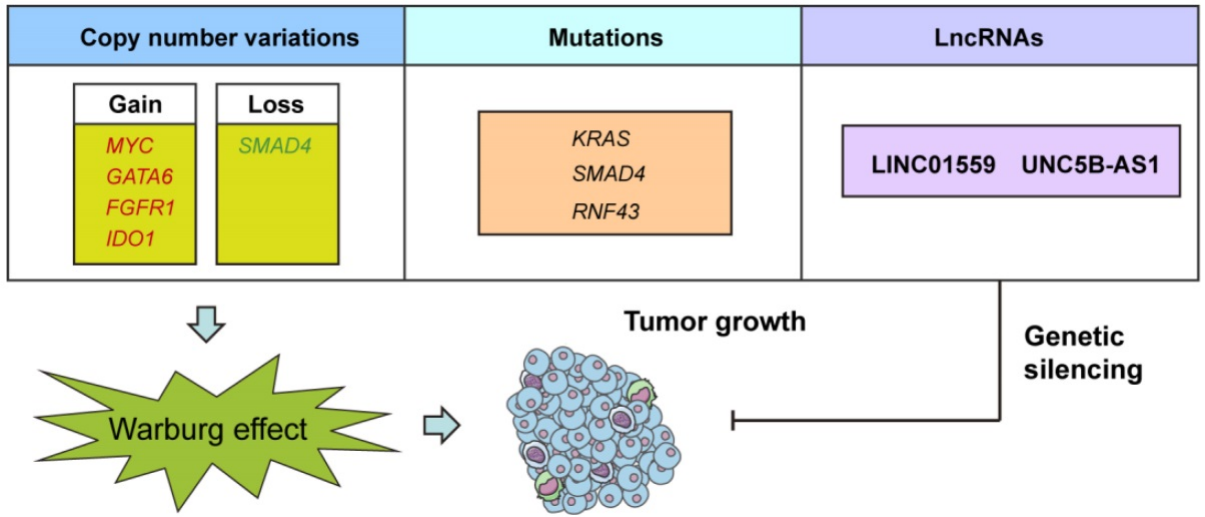
** A schematic diagram shows genomic and lncRNA landscapes of aerobic glycolysis in PDAC.** Gene copy number variations (CNVs) in *MYC*, *GATA6*, *FGFR1*, *IDO1*, and *SMAD4*, gene mutations in *KRAS*, *SMAD4*, and *RNF43*, and dysregulation of LncRNAs, especially LINC01559 and UNC5B-AS1 were closely associated with PDAC glycolysis. Knockdown of LINC01559 or UNC5B-AS1 significantly inhibited tumor growth in PDAC.

In the current study, we made a number of important observations concerning genomic alterations and lncRNAs in the glycolytic phenotype in PDAC (**Fig. 7**). First, we found that several CNVs and mutations preferentially enriched in glycolysis-low or glycolysis-high samples. Second, we identified, many previously unstudied lncRNA as being associated with PDAC glycolysis and upregulated in PDAC tissues. Third, we found that inhibition of LINC01559 or UNC5B-AS1 expression resulted in decreased glycolysis and PDAC cell proliferation. To the best of our knowledge, this is the first report of deciphering any lncRNA involved in the regulation of aerobic glycolysis in PDAC.

CNV profiling of human tumors has uncovered recurrent patterns of DNA amplifications and deletions across diverse cancer types. Compelling evidence revealed that metabolic stress acts as a selective pressure underlying the recurrent CNAs observed in human cancers 27. In this study, our result highlights the previous unprecedented regulatory role of GATA6, FGFR1, IDO1, and SMAD4 in the metabolic reprogramming of PDAC. Actually, GATA6 has been reported to direct hepatocellular carcinoma cells to glycolytic metabolism and fosters tumorigenicity, self-renewal and metastasis by transcriptional regulation of PKM2 expression 28. Aberrant activation of the FGFR1 signaling pathway is sufficient to enhance the Warburg effect through differential regulation of LDHA and LDHB in prostate cancer 29. Interestingly, IDO1 plays important roles in maintaining the pluripotency of primed human embryonic stem cells by upregulating glycolysis. Moreover, SMAD4 promotes diabetic nephropathy by reducing glycolysis via direct interaction with PKM2 30. However, whether aerobic glycolysis is regulated by these CNVs in PDAC warrants further investigation. In addition, detailed functional and mechanism characterization are encouraged to verify these highlights.

In PDAC, *KRAS* mutation is critical to control tumor metabolism through promotion of glucose uptake and channeling of glucose intermediates into the hexosamine biosynthesis and the nonoxidative arm of pentose phosphate pathway 16. Consistently, we confirmed the driver role of *KRAS* mutation in glucose metabolism. Moreover, our findings emphasize the importance of frequently mutated genes *SMAD4*, *GNAS*, *RNF43*, *TGFBR2,* and* PBRM1* in modulating PDAC glycolytic phenotypes. Recently, Liang et al. showed that the glycolytic enzyme PGK1 is transcriptionally repressed by SMAD4 and SMAD4 inactivation in PDAC induces PGK1 upregulation to enhance glycolysis and aggressive tumor behaviors 31. Specifically, SMAD4 may also interact with hypoxia-inducible factor 1α (HIF1α) to regulate target genes to suppress a glycolytic phenotype 32. Additionally, PBRM1 is known to be important for driving renal clear cell carcinoma through the regulation of hypoxia response genes, PI3K signaling, and glucose uptake 33. These studies support our findings regarding the recurrent gene mutations involved in glycolytic metabolism. However, additional verification should be carried out to yield insight into these mutations shaping tumor glycolysis.

LINC01559 has been identified as a potential non-invasive biomarker of renal cell carcinoma and is reported to accelerate pancreatic cancer cell proliferation and invasion through enhancing YAP activity, and UNC5B-AS1 is associated with tumourigenesis and metastasis of papillary thyroid cancer 21, 23, 34. Our results suggest that LINC01559 and UNC5B-AS1 are novel regulators of aerobic glycolysis in PDAC. Given the close expression correlation between LINC01559 and UNC5B-AS1 and nearly all glycolytic genes (glucose transporter and glycolytic enzymes), we postulate a mechanism of chromatin organization and transcriptional regulation mediated by LINC01559 and/or UNC5B-AS1 to promote aerobic glycolysis. Consistent with this notion, genetic silencing of LINC01559 or UNC5B-AS1 led to transcriptional misregulation in cancer. Apart from LINC01559 and UNC5B-AS1, many differentially expressed LncRNAs were predicted to result in aerobic glycolysis, such as SH3PXD2A-AS1, SOX21-AS1, and FAM83A-AS1. Future studies may unravel the regulatory role of these candidates on the Warburg metabolism in PDAC.

In conclusion, our integrated analysis of the molecular landscape of PDAC aerobic glycolysis has yielded important insights into the biology of this deadly disease. Our observations raise the novel regulatory roles of recurrent somatic gene mutations and copy number alterations in PDAC metabolic reprogramming. In addition, targeting dysregulated lncRNAs, especially LINC01559 and UNC5B-AS1, may represent a potential therapeutic strategy by inhibiting aerobic glycolysis in PDAC.

## Supplementary Material

Supplementary figures and tables.

## Figures and Tables

**Table 1:** Differentially expressed glycolysis-related lncRNAs in PDAC

LncRNA	logFC	*P*-Value	LncRNA	logFC	*P*-Value	LncRNA	logFC	*P*-Value
FEZF1-AS1	3.07	1.39E-06	BX470102.1	2.15	9.96E-12	AC103702.2	2.00	0.001228
UNC5B-AS1	2.98	3.46E-14	AC093904.2	2.14	6.40E-09	FAM30A	-2.79	1.41E-09
AP002498.1	2.83	2.55E-08	AP000696.1	2.13	1.13E-06	LINC00402	-2.77	1.43E-10
AC002384.1	2.83	1.67E-09	AC105219.2	2.12	3.13E-08	AL139020.1	-2.67	1.20E-07
LINC01559	2.65	2.12E-09	AC005256.1	2.12	4.82E-07	AC002546.2	-2.65	1.51E-08
AP005233.2	2.52	1.40E-07	LINC00483	2.12	5.02E-05	LINC02397	-2.64	1.96E-08
CYMP-AS1	2.47	1.04E-05	MIR210HG	2.12	8.57E-12	LINC01781	-2.63	2.22E-07
SH3PXD2A-AS1	2.46	1.18E-11	AC068580.2	2.11	4.85E-08	LINC00494	-2.42	5.23E-08
SOX21-AS1	2.46	1.13E-06	AC004990.1	2.11	2.27E-08	LINC02273	-2.41	1.31E-11
FAM83A-AS1	2.44	3.83E-06	LINC01978	2.10	6.38E-11	AL365361.1	-2.41	7.99E-13
AC021218.1	2.40	7.02E-08	AL512328.1	2.10	1.67E-06	LINC00861	-2.41	2.10E-11
AL355388.1	2.37	6.46E-09	MAL2	2.10	9.35E-13	CADM3-AS1	-2.39	5.22E-11
AL049836.1	2.35	1.40E-10	TRIM31-AS1	2.10	2.46E-11	AL928742.1	-2.37	4.12E-07
AC130456.2	2.31	7.86E-09	AL365226.2	2.09	0.005693	IFNG-AS1	-2.35	2.36E-09
AC090164.2	2.31	2.38E-06	AC120498.4	2.07	8.10E-08	AL583785.1	-2.31	5.18E-09
AL355312.3	2.30	1.46E-09	SYNPR-AS1	2.07	3.49E-09	AC022239.2	-2.25	3.02E-06
LINC02323	2.29	7.72E-14	AC106900.2	2.06	3.65E-06	LINC02422	-2.17	3.31E-06
LINC00520	2.28	9.81E-08	AC024592.2	2.05	6.70E-11	AC022239.3	-2.14	3.74E-06
AFAP1-AS1	2.28	2.87E-05	AC012317.1	2.05	3.52E-07	AL161781.2	-2.14	1.00E-07
AC005550.3	2.25	0.000365	LINC02041	2.04	3.06E-09	AL133467.1	-2.10	1.85E-10
C5orf66-AS1	2.22	1.78E-06	AC008687.2	2.04	9.28E-08	U62631.1	-2.10	1.13E-07
AC008687.3	2.22	3.28E-08	KRT7-AS	2.02	7.29E-11	LINC01215	-2.07	5.59E-06
SLCO4A1-AS1	2.22	2.63E-09	AC114488.1	2.02	3.43E-11	AL512638.2	-2.06	1.39E-09
AC093904.4	2.21	3.41E-07	LINC02320	2.02	3.39E-08	AP000894.2	-2.05	5.39E-06
AC004009.1	2.18	1.59E-05	MUC2	2.01	0.022248	AC006369.1	-2.05	6.67E-07
BLACAT1	2.15	1.26E-09	AL596223.1	2.01	5.64E-12			

## Abbreviations

PDAC: Pancreatic ductal adenocarcinoma
SUVmax: maximum standard uptake value
SCNAs: somatic copy number alterations
LncRNA: long non-coding RNA
GEO: Gene Expression Omnibus
TCGA: The Cancer Genome Atlas
ECAR: extracellular acidification rate
