# Supplementary material for: Deciphering the genomic and lncRNA landscapes of aerobic glycolysis identifies potential therapeutic targets in pancreatic cancer
Source: Int J Biol Sci. 2021 Jan 1;17(1):107–18. doi: 10.7150/ijbs.49243 (PMC7757027; doi:10.7150/ijbs.49243)
Supplement: Supplementary file 1 — Supplementary figures and tables. [file ijbsv17p0107s1.pdf]

Supplementary Information

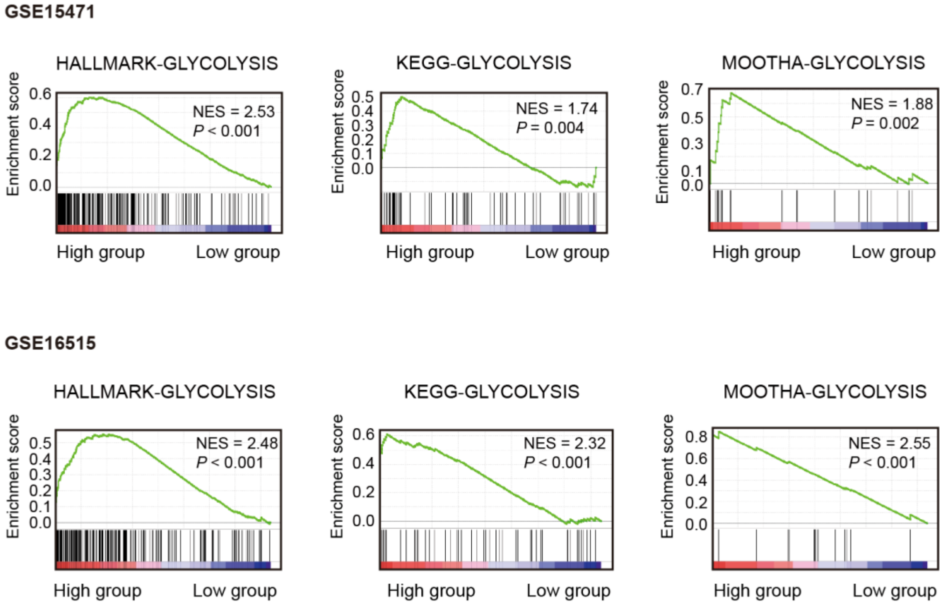

Supplementary Figure 1. Gene set enrichment analysis on three glycolysis gene sets across the glycolysis-low and glycolysis-high samples. Data were derived from the GEO datasets GSE15471 and GSE16515. NES, normalized enrichment score (NES); false discovery rate (FDR) was set at 0.25.

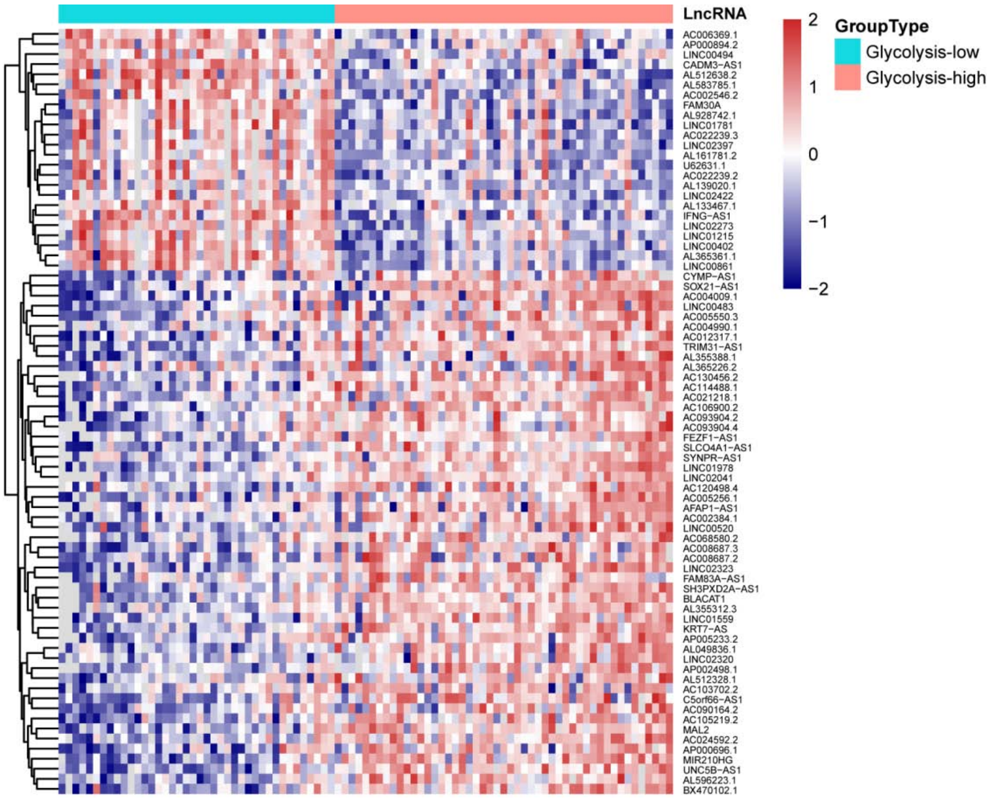

Supplementary Figure 2. Heatmap showed differentially expressed lncRNAs between glycolysis-low and glycolysis-high groups.

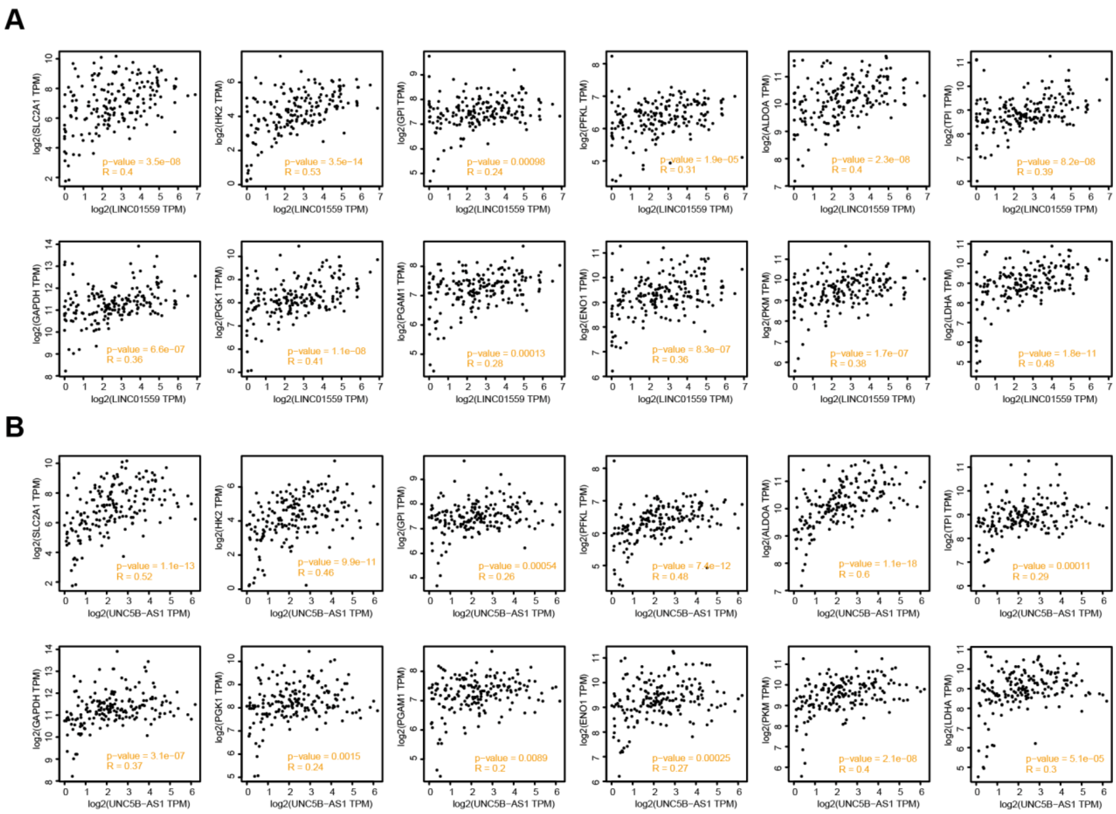

Supplementary Figure 3. Correlation analysis of the link between LINC01559 (**A**) and UNC5B-AS1 (**B**) expression and level of glycolytic components in PDAC. Data were acquired from TCGA cohort.

Supplementary Table 1: Group information

Glycolysis-high group	Glycolysis-Low group
TCGA-2J-AAB4-01	TCGA-2J-AAB9-01
TCGA-2J-AAB8-01	TCGA-2J-AABF-01
TCGA-2J-AABA-01	TCGA-2J-AABO-01
TCGA-2J-AABH-01	TCGA-2J-AABR-01
TCGA-2J-AABK-01	TCGA-2L-AAQM-01
TCGA-2L-AAQA-01	TCGA-3A-A9I5-01
TCGA-2L-AAQI-01	TCGA-3A-A9IN-01
TCGA-2L-AAQJ-01	TCGA-3A-A9IX-01
TCGA-2L-AAQL-01	TCGA-FB-A4P5-01
TCGA-3A-A9IH-01	TCGA-FB-A5VM-01
TCGA-3A-A9IU-01	TCGA-H8-A6C1-01
TCGA-3A-A9IZ-01	TCGA-HZ-7918-01
TCGA-F2-A44G-01	TCGA-HZ-7920-01
TCGA-F2-A8YN-01	TCGA-HZ-7923-01
TCGA-FB-A545-01	TCGA-HZ-8001-01
TCGA-FB-A78T-01	TCGA-HZ-8002-01
TCGA-FB-AAPP-01	TCGA-HZ-8003-01
TCGA-FB-AAPQ-01	TCGA-HZ-A4BH-01
TCGA-FB-AAPZ-01	TCGA-HZ-A4BK-01
TCGA-FB-AAQ1-01	TCGA-IB-7649-01
TCGA-FB-AAQ6-01	TCGA-IB-7888-01
TCGA-H6-A45N-01	TCGA-IB-7891-01
TCGA-HV-A5A6-01	TCGA-IB-8126-01
TCGA-IB-7885-01	TCGA-IB-A5ST-01
TCGA-IB-7889-01	TCGA-IB-AAUM-01
TCGA-IB-A5SO-01	TCGA-IB-AAUP-01
TCGA-IB-A5SP-01	TCGA-IB-AAUS-01
TCGA-IB-A6UF-01	TCGA-IB-AAUV-01
TCGA-IB-A7LX-01	TCGA-IB-AAUW-01
TCGA-IB-A7M4-01	TCGA-LB-A7SX-01
TCGA-IB-AAUN-01	TCGA-LB-A8F3-01
TCGA-IB-AAUO-01	TCGA-LB-A9Q5-01
TCGA-IB-AAUU-01	TCGA-Q3-A5QY-01

TCGA-M8-A5N4-01	TCGA-RB-AA9M-01
TCGA-OE-A75W-01	TCGA-US-A774-01
TCGA-PZ-A5RE-01	TCGA-US-A77J-01
TCGA-Q3-AA2A-01	TCGA-XD-AAUH-01
TCGA-RB-A7B8-01	TCGA-XD-AAUI-01
TCGA-S4-A8RM-01	TCGA-XN-A8T5-01
TCGA-S4-A8RO-01	TCGA-Z5-AAPL-01
TCGA-S4-A8RP-01	
TCGA-US-A776-01	
TCGA-US-A779-01	
TCGA-US-A77E-01	
TCGA-US-A77G-01	
TCGA-XD-AAUL-01	
TCGA-YB-A89D-01	
TCGA-YH-A8SY-01	
TCGA-YY-A8LH-01	

Supplementary Table 2: Significantly reoccurring amplification in glycolysis-high group

cytoband	8q24.21	18q11.2	18p11.31	7q21.3	1p12	8p11.21
q value	0.0019209	0.0019209	0.046066	0.071368	0.18265	0.21166
residual q value	0.0019209	0.0019209	0.046066	0.071368	0.18265	0.21166
wide peak boundaries	chr8:127949878<br>-129253822	chr18:19027829<br>-21027113	chr18:1607584<br>-4072947	chr7:97511362<br>-99231825	chr1:116111930<br>-149882013	chr8:37287392<br>-41745533
genes in wide peak	hsa-mir-1208	hsa-mir-1-2	TGIF1	NPTX2	hsa-mir-3118-3	hsa-mir-486
	hsa-mir-1207	hsa-mir-320c-1	MYOM1	OCM2	hsa-mir-3118-2	ADRB3
	hsa-mir-1205	GATA6	DLGAP1	TRRAP	hsa-mir-3118-1	ANK1
	hsa-mir-1204	RBBP8	LPIN2	BUD31	hsa-mir-942	ADAM3A
	MYC	SNRPD1	NDC80	ATP5J2	hsa-mir-320b-1	EIF4EBP1
	POU5F1B	MIB1	MYL12A	ARPC1B	ATP1A1	FGFR1
	PVT1	CTAGE1	SMCHD1	ARPC1A	BCL9	ADAM2
	LOC727677	GREB1L	METTL4	CPSF4	CASQ2	IDO1
	MIR1205	TMEM241	EMILIN2	PDAP1	CD2	SFRP1
	MIR1206	CABLES1	MYL12B	LMTK2	CD58	STAR
	MIR1207	ESCO1	LOC201477	ZKSCAN5	FCGR1A	TACC1
	MIR1204	ABHD3	CBX3P2	BRI3	FCGR1B	ADAM18
	MIR1208	MIR1-2	FLJ35776	TECPR1	FMO5	ADAM9
	PCAT1	MIR133A1	LOC727896	PTCD1	GJA5	ASH2L
		MIR320C1		BAIAP2L1	GJA8	BAG4
		MIR4741		SMURF1	HMGCS2	ERLIN2
				ZNF655	HSD3B1	PROSC
				ZNF394	HSD3B2	DDHD2
				MYH16	IGSF3	GPR124
				BHLHA15	NHLH2	LSM1
				ZNF498	NOTCH2	GOLGA7
				FAM200A	PDZK1	WHSC1L1
				TMEM130	PRKAB2	BRF2
				ZNF789	PTGFRN	C8orf4
				MGC72080	TBX15	PLEKHA2
				KPNA7	HIST2H2AA3	ZMAT4
				LOC100289187	HIST2H2AC	ZNF703
				MIR3609	HIST2H2BE	RAB11FIP1
				ATP5J2-PTCD1	HIST2H4A	TM2D2
					TTF2	GINS4
					ITGA10	PPAPDC1B
					PEX11B	GOT1L1
					CD101	AGPAT6
					SEC22B	LETM2
					CHD1L	NKX6-3
					PDE4DIP	IDO2
					SV2A	HTRA4

RBM8A	ADAM32
WARS2	ADAM5P
PIAS3	RNF5P1
POLR3C	C8orf86
TXNIP	MIR486
WDR3	LOC728024
MAN1A2	LOC100130964
ADAM30	
CD160	
NBPF14	
PHGDH	
RNF115	
BOLA1	
HAO2	
ACP6	
GPR89B	
GDAP2	
FAM46C	
SLC22A15	
FAM91A2	
VTCN1	
TRIM45	
VANGL1	
REG4	
POLR3GL	
ATP1A1OS	
ZNF697	
GNRHR2	
MAB21L3	
HIST2H3C	
LIX1L	
HSD3BP4	
HFE2	
ANKRD35	
PPIAL4A	
PDIA3P	
NBPF11	
NUDT17	
SPAG17	
NBPF15	
ANKRD34A	
HIST2H2AB	
HIST2H3A	
HIST2H2BC	

	HIST2H2BA
	NBPF7
	LOC375010
	NOTCH2NL
	FLJ39739
	LOC388692
	NBPF9
	HIST2H2BF
	HIST2H4B
	LOC644242
	PPIAL4G
	PPIAL4D
	LOC645166
	EMBP1
	SRGAP2P2
	PPIAL4B
	LOC653513
	GPR89A
	PPIAL4C
	HIST2H3D
	FAM72B
	HIST2H2AA4
	FAM72D
	LOC728855
	LOC728875
	NBPF24
	GPR89C
	NBPF16
	PDZK1P1
	PPIAL4F
	LOC728989
	PPIAL4E
	PFN1P2
	MIR942
	LOC100130000
	NBPF10
	FCGR1C
	LOC100286793
	LOC100289211
	MIR320B1

Supplementary Table 3: Significantly reoccurring deletion in glycolysis-high group

cytoband	9p21.3	18q21.2
q value	1.63E-18	3.90E-08
residual q value	1.63E-18	3.90E-08
wide peak boundaries	chr9:21846441-22449908	chr18:48472083-51155767
genes in wide peak	CDKN2A	DCC
	CDKN2B	SMAD4
	C9orf53	MEX3C
	CDKN2B-AS1	ELAC1
		LOC100287225

Supplementary Table 4: Significantly reoccurring amplification in glycolysis-low group

cytoband	19q13.2	17q21.33
q value	0.0051148	0.19884
residual q value	0.0051148	0.19884
wide peak boundaries	chr19:33673133-41408864	chr17:47875865-50048922
genes in wide peak	hsa-mir-641	CHAD
	ACTN4	COL1A1
	AKT2	DLX3
	APLP1	DLX4
	ATP4A	ITGA3
	BLVRB	NME1
	CAPNS1	NME2
	CD22	PDK2
	CEBPA	SGCA
	CEBPG	ABCC3
	TBCB	CACNA1G
	CLC	SPAG9
	COX6B1	TOB1
	COX7A1	KAT7
	CYP2A6	UTP18
	CYP2A7	MRPL27
	ECH1	LUC7L3
	FBL	MBTD1
	ETV2	LINC00483
	GPI	EPN3
	FFAR1	RSAD1
	FFAR3	LRRC59
	FFAR2	CA10
	HNRNPL	XYLT2
	HPN	SPATA20
	LGALS4	ACSF2
	LGALS7	MYCBPAP
	LRP3	PPP1R9B
	MAG	ANKRD40
	MAP3K10	WFIKKN2
	NFKBIB	EME1
	NPHS1	TMEM92
	PEPD	SAMD14
	FXYD1	LOC253962
	FXYD3	TAC4
	POLR2I	LOC284080
	PSMC4	HILS1
	PSMD8	LOC400604
	MRPS12	NME1-NME2
	RPS16	FLJ45513
	RYR1	
	SCN1B	
	SNRPA	
	SUPT5H	
	TYROBP	
	USF2	
	ZFP36	
	ZNF146	
	MIA	
	DPF1	
	LTBP4	
	FCGBP	
	DYRK1B	
	NUMBL	

KCNK6
GMFG
KIAA0355
MLL4
UBA2
PAK4
TMEM147
SPINT2
DLL3
HCST
UPK1A
MAP4K1
ZFP30
SIRT2
CYP2G1P
SIPA1L3
HAUS5
PLD3
ZNF345
CLIP3
LSM14A
GAPDHS
ZBTB32
EIF3K
LGALS13
SERTAD3
SERTAD1
ZNF571
LSR
FXYD7
FXYD5
RAB4B
PAF1
SARS2
SAMD4B
MED29
PSENEN
ZNF302
LIN37
SLC7A10
LGALS14
LRFN1
GRAMD1A
ZFP14
ZNF529
PRX
SPTBN4
HAMP
CATSPERG
PRODH2
C19orf33
CHST8
PLEKHG2
KCTD15
RBM42
LRFN3
IGFLR1
ADCK4
CNTD2
LOC80054
ITPKC

WDR87
KIRREL2
PDCD2L
ZNF527
ZNF607
NFKBID
ZNF382
ZNF566
ALKBH6
ZNF30
YIF1B
ZNF461
ZNF585B
TIMM50
SHKBP1
DMKN
PPP1R14A
EGLN2
FBXO17
ARHGAP33
RASGRP4
ZNF573
EID2B
WTIP
ZNF792
HSPB6
RINL
FBXO27
C19orf47
HIPK4
ZNF420
ZNF565
FAM98C
CAPN12
LGALS16
TTC9B
ZNF599
FAM187B
C19orf55
ZNF569
ZNF570
ZNF567
ZNF383
ZNF781
EID2
ZNF780B
LGI4
C19orf46
ZNF540
ZNF585A
GGN
THAP8
U2AF1L4
IL28A
IL28B
IL29
ZNF780A
C19orf54
SCGB2B2
WDR62
ZFP82

LOC284408
LOC284412
HKR1
ZNF181
ZNF260
ZNF546
ZNF850
NCCRP1
SYCN
LEUTX
SELV
SBSN
ZNF829
ZNF568
KRTDAP
ZNF790
ZNF793
PAPL
SPRED3
LOC400685
LGALS17A
LOC643669
SCGB1B2P
SDHAF1
LOC644189
LOC644554
LGALS7B
MIR641
LOC728752
LOC100128675
LOC100128682
LOC100129935
SCGB2B3P
LOC100134317
LOC100506469
LOC100506930
LOC100507433
MIA-RAB4B
RAB4B-EGLN2
MIR4530
LOC100631378
LOC100652909

Supplementary Table 5: Significantly reoccurring deletion in glycolysis-low group

cytoband	9p21.3
q value	8.00E-06
residual q value	8.00E-06
wide peak boundaries	chr9:21785661-24867805
genes in wide peak	CDKN2A
	CDKN2B
	ELAVL2
	MTAP
	C9orf53
	DMRTA1
	FLJ35282
	CDKN2B-AS1

Supplementary Table 6: Differentially expressed gene upon LINC01559 knockdown in BxPC-3 cells

Gene name	log_2FC	P-value
HIST2H3C	-1.00264	1.52E-05
OLFM4	-1.00355	9.03E-05
LIMS1	-1.00445	6.59E-05
RBP1	-1.0117	7.83E-09
PRSS2	-1.01189	0.000171
SNX12	-1.01436	9.02E-05
SERPINB9	-1.02357	4.33E-15
NOTCH2	-1.03415	2.05E-15
TENT5B	-1.038	0.000163
MRPL51	-1.0469	3.70E-11
SLC16A2	-1.05043	1.17E-06
CAPZA1	-1.06083	6.85E-18
HDHD5	-1.0614	2.35E-07
ZPR1	-1.08497	7.95E-13
GRB2	-1.10275	4.26E-10
PRSS22	-1.10311	4.10E-10
GPR180	-1.10404	2.68E-10
NIP7	-1.11065	1.18E-07
PTGS1	-1.11827	8.67E-14
ARF3	-1.12166	5.87E-20
DNAJC25-GNG10	-1.13389	1.52E-05
CYSRT1	-1.16756	2.33E-10
ARF1	-1.17311	9.34E-07
LIPG	-1.1754	2.38E-08
HIST2H3A	-1.19187	6.17E-05
PPT2	-1.19411	1.06E-11
DUSP2	-1.20065	5.63E-05
GEN1	-1.20123	0.000119
C17orf80	-1.20311	1.68E-09
ZBTB9	-1.22775	0.00048
ANKRD1	-1.2542	1.29E-13
CA12	-1.25892	5.84E-14
AC027644.4	-1.26558	5.78E-06
ACTRT3	-1.28294	1.06E-06
TOMM40L	-1.32503	9.03E-08
AC068580.4	-1.33024	0.000933
AHNAK2	-1.36294	9.76E-23

IFIT2	-1.36877	1.36E-25
KRT80	-1.41561	1.66E-13
IVL	-1.41973	4.58E-08
JPT2	-1.45394	0.000175
LFNG	-1.45657	3.77E-16
VGLL1	-1.46185	9.35E-08
APAF1	-1.46285	1.05E-11
IGFL1	-1.49233	1.56E-07
TMEM265	-1.49927	3.17E-05
MRFAP1	-1.50118	2.20E-06
SHROOM2	-1.5628	2.16E-06
NSRP1	-1.58505	2.07E-17
MT-ATP6	-1.78883	8.62E-07
MMP9	-2.29357	8.11E-14
CDH5	-2.74282	1.40E-30
AP002990.1	-2.94749	2.42E-11
UGT1A3	-5.4805	8.74E-09
BLOC1S5-TXNDC5	-12.1276	2.92E-06

Supplementary Table 7: Differentially expressed gene upon UNC5B-AS1 knockdown in BxPC-3 cells

Gene name	log_2FC	P-value
SELENOF	-1.00573	3.20E-06
CLDN12	-1.0076	0.000532
LMBR1	-1.00791	7.09E-10
AP001458.2	-1.01301	0.001112
FGFR3	-1.01742	2.50E-07
AC006486.1	-1.0184	0.002407
HTR7	-1.0208	1.42E-05
RAB12	-1.02122	1.52E-09
MTCH1	-1.02236	4.87E-14
UNC5B	-1.02271	1.30E-07
ADH7	-1.02926	0.000334
PYGL	-1.03508	2.38E-14
NUDCD2	-1.0424	3.93E-10
PPP1R12B	-1.04286	0.00022
PIMREG	-1.05021	0.000431
LYNX1	-1.05117	8.61E-06
NXPH4	-1.05266	0.000255
EYA2	-1.05724	2.47E-09
NUDT1	-1.05966	6.01E-07
ARFGAP2	-1.06557	7.99E-12
NUDT8	-1.06819	1.92E-06
LDLRAP1	-1.07062	4.53E-05
ALDH3A2	-1.07348	2.38E-07
VAMP5	-1.08108	0.000139
LIME1	-1.08236	2.57E-05
AXL	-1.08293	1.59E-13
TNFAIP8L1	-1.08844	2.49E-06
ZNF641	-1.08991	0.002117
SULF2	-1.09036	1.68E-12
ETV7	-1.09037	4.40E-07
CELSR2	-1.10229	3.56E-13
C16orf58	-1.10479	4.79E-12
DHRS2	-1.10561	3.49E-10
MEGF6	-1.10818	6.08E-07
DYRK3	-1.11176	6.83E-07
FZD5	-1.11224	4.28E-10
KRT5	-1.12248	2.45E-15

KRT80	-1.12454	1.72E-08
IL6R	-1.13225	1.30E-10
ADIRF	-1.13469	2.45E-07
ARPIN	-1.13773	1.34E-11
HIST1H2AG	-1.14251	2.13E-05
CDKN2C	-1.14312	6.77E-08
FEM1A	-1.1444	2.96E-12
S100A3	-1.15269	2.89E-06
C2CD4A	-1.15351	0.000324
LAMP1	-1.15605	1.10E-11
DHFR	-1.17178	0.000756
SPINK5	-1.17339	4.02E-10
LBR	-1.17416	1.08E-15
KLK11	-1.17846	2.36E-13
HSPE1-MOB4	-1.1859	0.002018
LY6G5B	-1.18995	0.000253
ARMT1	-1.19384	0.000319
STX6	-1.19717	3.91E-05
PLEKHA4	-1.20812	1.11E-10
HIST1H2BJ	-1.21298	7.60E-06
AC011448.1	-1.22103	0.000234
HIST1H3H	-1.22258	1.16E-05
EIF2S3B	-1.23899	0.002215
RHPN2	-1.25867	6.61E-20
C17orf80	-1.26249	8.13E-08
CST2	-1.27817	0.000102
NOXA1	-1.28985	0.000265
PRRG1	-1.29117	0.000507
TRNP1	-1.30214	4.43E-07
SMAD6	-1.30293	1.80E-07
HIST1H2AI	-1.31024	0.002121
RIBC2	-1.32125	3.89E-06
TGIF2	-1.32133	0.000295
B9D2	-1.33855	4.12E-06
SH3TC1	-1.37381	1.30E-11
KLK13	-1.40372	1.45E-09
S100A4	-1.42249	3.43E-09
LGR6	-1.50264	9.68E-11
GEN1	-1.5115	2.23E-06
PRSS1	-1.52978	1.15E-07

HIST2H3A	-1.53778	2.96E-08
METTL7A	-1.55426	8.49E-06
HIST2H3C	-1.59106	7.22E-09
SYT12	-1.59342	1.16E-11
TNNI2	-1.60126	1.88E-07
ADNP	-1.68448	3.29E-05
ALDH3A1	-1.70672	1.15E-28
RAMP1	-1.73969	1.04E-07
CAVIN2	-1.77003	4.81E-12
ADAT3	-1.81472	4.04E-05
AC116366.3	-1.88327	0.001948
LFNG	-1.97827	2.83E-19
CCL17	-2.02042	0.000159
PDIA5	-2.10102	3.10E-29
CCR4	-2.17394	5.44E-09
CALML5	-2.17454	1.21E-05
UBN1	-2.49337	4.83E-69
CDH5	-2.62302	1.54E-19
PRSS2	-2.6432	8.69E-16
AC007240.1	-3.97391	7.00E-06
AC138811.2	-4.49862	1.85E-05
UGT1A3	-6.12876	2.05E-09
